# Supplementary figures and images for: Establishment and validation of an individualized macrophage-related gene signature to predict overall survival in patients with triple negative breast cancer
Source: PeerJ. 2021 Nov 23;9:e12383. doi: 10.7717/peerj.12383 (PMC8621725; doi:10.7717/peerj.12383)

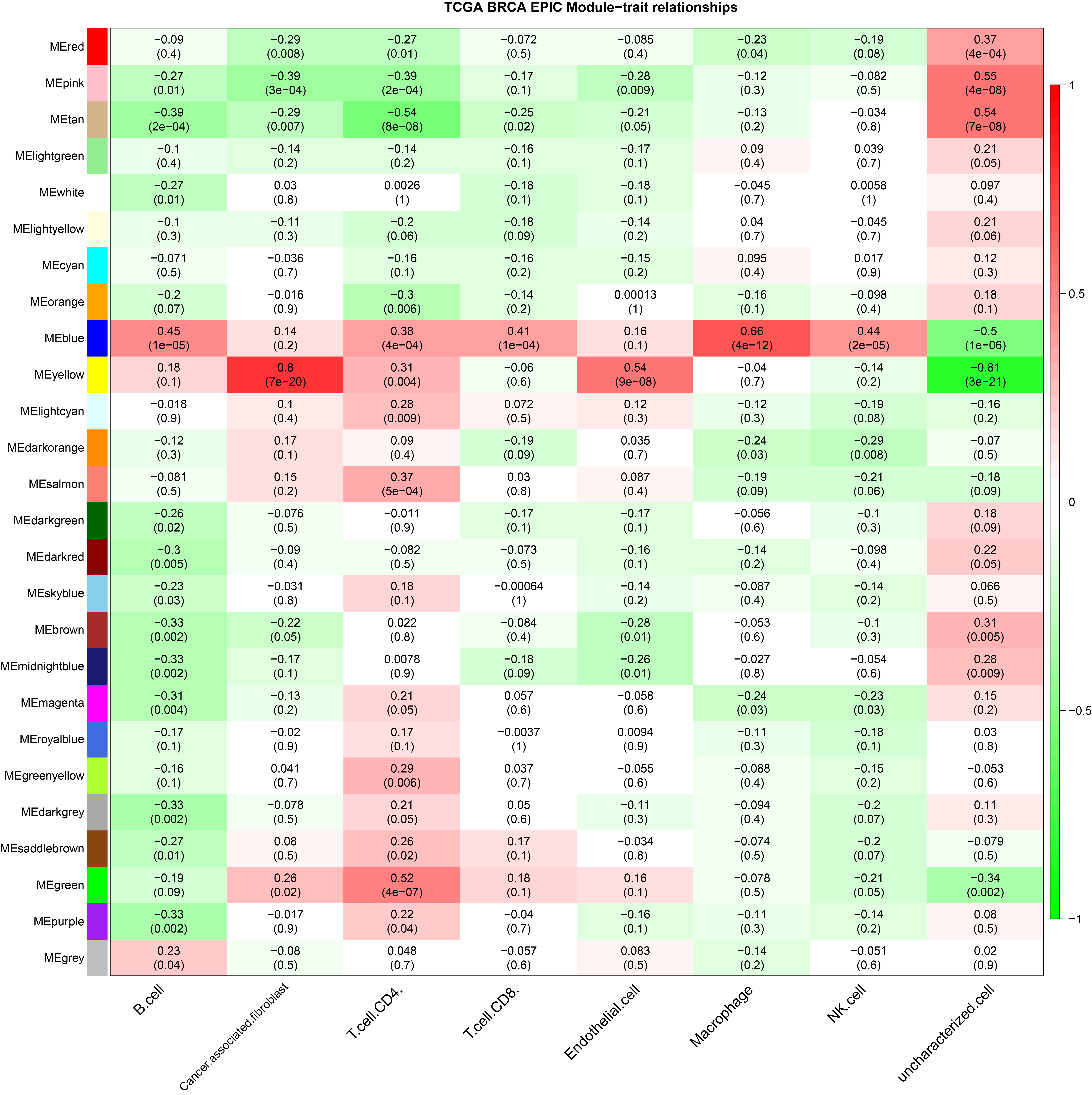

Supplement: Supplemental Information 1 [file peerj-09-12383-s001.png]

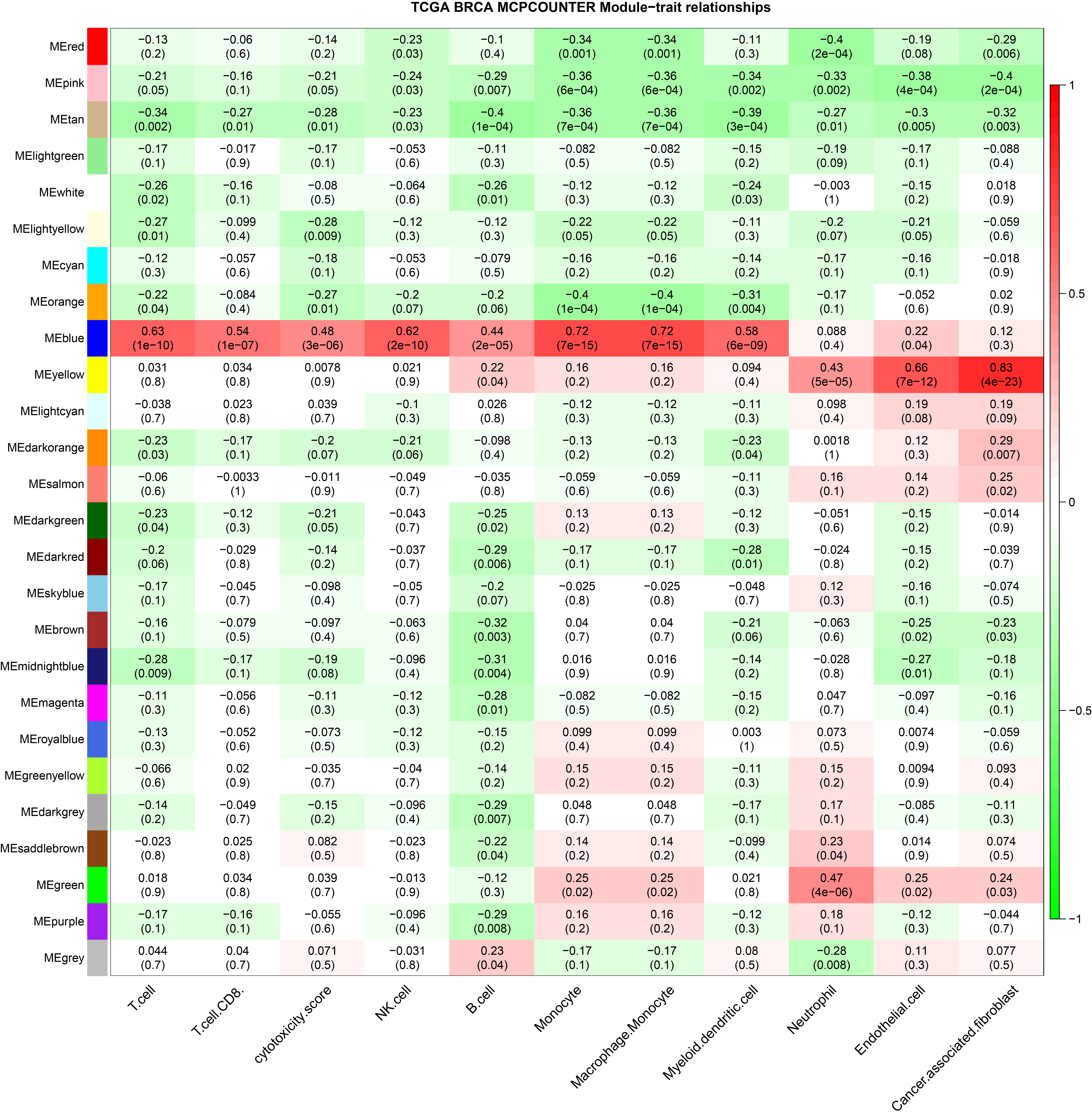

Supplement: Supplemental Information 2 [file peerj-09-12383-s002.png]

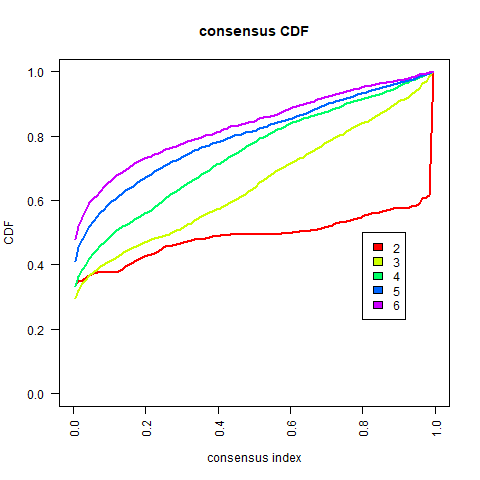

Supplement: Supplemental Information 6 [file peerj-09-12383-s006.png]

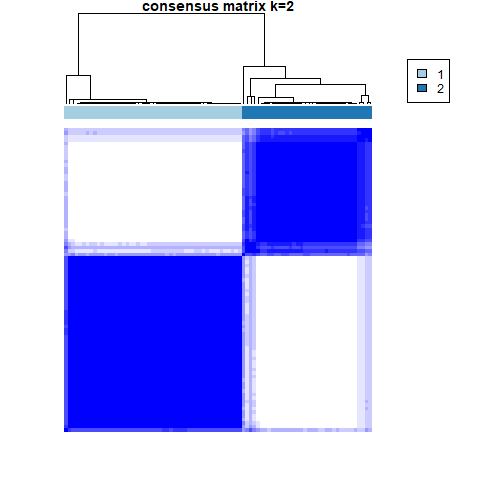

Supplement: Supplemental Information 7 [file peerj-09-12383-s007.png]
