# Supplementary material for: Establishment and validation of an individualized macrophage-related gene signature to predict overall survival in patients with triple negative breast cancer
Source: PeerJ. 2021 Nov 23;9:e12383. doi: 10.7717/peerj.12383 (PMC8621725; doi:10.7717/peerj.12383)
Supplement: Supplemental Information 8 [file peerj-09-12383-s008.docx]

| TableS1. The basic characteristics of the patients in GSE76124 | |
| --- | --- |
| Characteristic | Freq |
| Race , No. (%) |  |
| Asian | 3 (2%) |
| Asian/Pacific Islander | 4 (2%) |
| Caucasian | 184 (96%) |
| Grade , No. (%) |  |
| Moderately Differentiated | 51 (31%) |
| Poorly Differentiated | 107 (66%) |
| Well Differentiated | 4 (2%) |
| Tumor_Size , No. (%) |  |
| <=2 | 34 (19%) |
| >5 | 12 (7%) |
| 2_5 | 137 (75%) |
| Positive_Nodes , No. (%) |  |
| >=10 | 10 (7%) |
| 0 | 73 (49%) |
| 1_3 | 49 (33%) |
| 4_9 | 17 (11%) |
| BMI,median (interquartile range) | 27 (24to31) |
| TNBC_subtype , No. (%) |  |
| Basal-Like Immune-Activated (BLIA) | 54 (28%) |
| Basal-Like Immune-Suppressed (BLIS) | 58 (30%) |
| Luminal-AR (LAR) | 36 (18%) |
| Mesenchymal (MES) | 47 (24%) |
| Age, mean±SD | 54.58±12.68 |
| T_Stage , No. (%) |  |
| T1 | 34 (17%) |
| T2 | 137 (70%) |
| T3 | 12 (6%) |
| T4 | 9 (5%) |
| TX | 3 (2%) |
| N_Stage , No. (%) |  |
| N0 | 73 (37%) |
| N1 | 49 (25%) |
| N2 | 17 (9%) |
| N3 | 10 (5%) |
| NX | 46 (24%) |
| M_Stage , No. (%) |  |
| M0 | 142 (73%) |
| M1 | 2 (1%) |
| MX | 51 (26%) |
