# Supplementary material for: Establishment and validation of an individualized macrophage-related gene signature to predict overall survival in patients with triple negative breast cancer
Source: PeerJ. 2021 Nov 23;9:e12383. doi: 10.7717/peerj.12383 (PMC8621725; doi:10.7717/peerj.12383)
Supplement: Supplemental Information 9 [file peerj-09-12383-s009.docx]

| TableS2. The basic characteristics of the patients in GSE103091 | |
| --- | --- |
| Characteristic | Freq |
| Age, mean±SD | 56.97±12.8 |
| Metastasis_status , No. (%) |  |
| non-Metastasis | 76 (71%) |
| Metastasis | 31 (29%) |
| MFS(days), median (interquartile range) | 2025 (1000.5 to 2996 ) |
| OS(days), median (interquartile range) | 2032 (1271.5 to 2996 ) |
| OS_status , No. (%) |  |
| Alive | 78 (73%) |
| Death | 29 (27%) |
